# Learning Preferences and Characteristics of Generation Z Students in Pediatric Nursing, Midwifery, Physiotherapy, Occupational Therapy, Radiology Assistance, and Paramedicine: Protocol for a Scoping Review

**DOI:** 10.2196/88232

**Published:** 2026-04-29

**Authors:** Iva Volešová, Daniela Javornická, Lenka Vrlová, Oluwakemi Rachael Adeboye, Eva Prušová, Petra Kašparová, Helena Kisvetrová, Annamaria Bagnasco, Mary Steen, Alison Steven

**Affiliations:** 1 Doctoral Studies Office Faculty of Health Sciences Palacký University Olomouc Olomouc, Olomoucký Czech Republic; 2 School of Healthcare and Nursing Sciences Faculty of Health and Wellbeing Northumbria University Newcastle, England United Kingdom; 3 Department of Nursing Faculty of Health Sciences, Palacký University Olomouc Olomouc, Olomoucký Czech Republic; 4 Department of Health Sciences University of Genoa Genoa, Liguria Italy; 5 Faculty of Health Sciences Faculty of Health Sciences Curtin University Perth, Western Australia Australia; 6 Department of Nursing and Midwifery Education and Research King Edward Memorial Hospital Perth, Western Australia Australia

**Keywords:** students, health care, learning, Generation Z, review, education, pediatric nursing, midwifery, physiotherapy, occupational therapy, radiology assistance, paramedicine

## Abstract

**Background:**

The global shortage of nursing and other health care professionals threatens the stability of health care systems. Generation Z (Gen Z), born between 1995 and 2010, represents a critical cohort for the future of the health care workforce. Educational institutions are adapting teaching strategies to address the learning preferences and expectations of this generation to teach effectively and prevent attrition. While some evidence exists regarding Gen Z nursing students, there is limited data on students in specialized health care fields, such as pediatric nursing, midwifery, physiotherapy, occupational therapy, radiology assistance, and paramedicine. Because students in these programs often focus on vulnerable patient populations and must navigate technologically complex environments, the development of targeted teaching methods should be informed by data from these cohorts.

**Objective:**

This scoping review aims to map the existing literature on the learning preferences of Gen Z students in selected health care disciplines.

**Methods:**

This review follows the PRISMA-ScR (Preferred Reporting Items for Systematic Reviews and Meta-Analyses Extension for Scoping Reviews) guidelines. The search strategy comprises 3 stages: a preliminary search in the PubMed database, a comprehensive search across multiple databases, and a reference list screening. Studies focused on Gen Z university students enrolled in pediatric nursing, midwifery, physiotherapy, occupational therapy, radiology assistance, and paramedicine programs were included. Studies that include other generations or disciplines such as medicine or general nursing were excluded. Data extraction and analysis follow the Joanna Briggs Institute guidelines, and both qualitative and quantitative findings will be synthesized narratively and descriptively. A broad research team provides multidisciplinary expertise, enabling a team approach and conflict resolution during the review process.

**Results:**

The search was conducted on December 18, 2024, in Scopus, CINAHL, Google Scholar, PubMed, Web of Science, PsycInfo, ProQuest Dissertations & Theses, and Academic Search Ultimate databases. The study was funded in December 2024. Screening of 590 unique records identified 2 (0.3%) eligible studies. The search was extended, and the Open Science Framework registration was updated. Searches in Embase and Emcare databases (July 2025) yielded 51 records. Following duplicate removal, 38 (74.5%) records underwent title and abstract screening. At the time of reporting, the research team will review the full texts and reference lists of 8 (21.1%) studies, to be followed by data extraction and analysis. Results are expected to be published by the end of July 2026.

**Conclusions:**

This review is expected to generate an evidence-informed overview of the learning preferences of Gen Z students across the selected health care disciplines. By identifying gaps and opportunities, the review aims to guide future research, curriculum design, and policy initiatives that strengthen the preparedness and retention of the upcoming health care workforce via tailored educational approaches.

**Trial Registration:**

OSF Registries 10.17605/OSF.IO/UMD7X; https://osf.io/umd7x/overview

**International Registered Report Identifier (IRRID):**

DERR1-10.2196/88232

## Introduction

The global shortage of nurses and other health care professionals is a growing concern [1-3]. The COVID-19 pandemic crisis and an aging health care workforce, with a projected 4.7 million retirements in the next decade, have contributed to a looming shortage of 13 million nurses [4]. Hence, recruiting and retaining Generation Z (Gen Z) students appears vital to the sustainability of the nursing and allied health professions workforce.

Gen Z, based on varied definitions as the cohort born approximately between 1995-1997 and 2010-2012 [5-7], is the first generation known to grow up fully immersed in a digital and technological environment, earning them the label of “digital natives” [8,9]. They are often described as ambitious, self-confident, and entrepreneurial, with a strong focus on personal growth and finding meaningful work [10,11]. This generation values advancement opportunities, skill development, and a positive work environment, leading to a tendency for frequent job changes when their expectations are not met [12]. Gen Z has been selected for this study as most current university students belong to this generation. While some members of Gen Z have already completed their degrees and are working in health care, the younger portion of the generation is either still in university or about to enter [13]. Educational institutions must prepare students by transferring cross-cutting competencies to effectively meet generational challenges and mitigate dropout from study programs due to disillusionment or mismatches between learning preferences and teaching strategies [14,15]. Education is a dynamic process that involves transferring continuously evolving knowledge and skills to the next generation of future professionals [16]. Professional education requires constant adaptation and updating of curricula to meet the specific needs of new generations of learners. Gen Z, having grown up alongside technology, exhibits learning styles and preferences that align closely with this digital phenomenon [17,18].

Shorey et al [7] emphasize the importance of creating safe learning environments that build students’ confidence. They also advocate for integrating digital storytelling and technology into classrooms. Similarly, Shatto and Erwin [19] highlight the shorter attention spans of Gen Z students, which should be considered in the classroom preparation of health care educators. They recommend incorporating mobile phones and tablets into classroom assignments to facilitate hands-on learning. Similarly, Mosca et al [20] recommend leveraging technology in classrooms to transition from teacher-centered to student-centered teaching. This adaptation could not only foster a more engaging learning environment but may also better prepare students from diverse professions for their future careers.

Isaacs et al [21] state that Gen Z health care students seek valuable work experience and practical skills that can be applied in their future careers. Similar to previous generations, these students value health care educators who not only support their academic growth but also foster their personal development. Emotional support, realistic expectations, and direct feedback [22,23] are essential components that Gen Z students expect from health care educators [24]. Research has shown that Gen Z differs significantly from millennials [25]. Millennials value clarity, practical application, and interactive teaching methods [26] more than previous generations. Gen Z appears more deeply immersed in technology than millennials, having grown up with constant global connectivity, social media, and instant information [27]. They appear to have shorter attention spans [28] and expect immediate answers. Compared with millennials, Gen Z seems more practical, career-focused, and financially driven, with a strong preference for self-learning and entrepreneurship [29,30]. They also appear to be less risk-averse and more politically disengaged, highlighting distinct generational differences [13].

Although nursing education has received considerable attention in recent years, there remains a lack of a deeper understanding of the nuanced and specific needs of students specializing in particular nursing fields (eg, pediatrics) and other allied health professions. Much less seems to be known about how Gen Z students experience learning in these specialist health professions that require distinct skill sets, clinical contexts, and professional identities. Disciplines such as pediatric nursing, midwifery, physiotherapy, occupational therapy, radiology assistance, and paramedicine involve high levels of patient interaction, emotional resilience, and technological competence. Understanding how Gen Z students approach learning in these specialized fields is crucial for designing curricula that foster both competence and professional commitment. A clearer understanding of these needs may also help reduce attrition rates and support workforce sustainability across the broader spectrum of health care professions.

## Methods

### Protocol Design

A scoping review was chosen because it is well-suited for mapping the breadth and diversity of evidence on a topic, particularly when the research area is broad, complex, or not well-defined [31]. Unlike systematic reviews, which focus on synthesizing specific evidence to address narrow research questions, scoping reviews are designed to allow exploration without predefined categories, providing flexibility to identify key concepts, gaps, and emerging themes [32]. This approach is considered ideal when the goal is to gain an overview of existing knowledge, clarify concepts, and inform future research directions [33]. The protocol for this scoping review has been registered on the Open Science Framework (OSF; [34]). The review will adhere to the PRISMA-ScR (Preferred Reporting Items for Systematic Reviews and Meta-Analyses Extension for Scoping Reviews) guidelines to ensure clarity in reporting and methodological rigor. The process will involve the following stages: (1) identify the research question; (2) identify relevant studies; (3) select studies; (4) chart the data; and (5) collate, synthesize, and report results.

### Stage 1: Research Question

The primary objective of this review is to identify any specific preferences of Gen Z university health care students in the fields of pediatric nursing, midwifery, physiotherapy, occupational therapy, radiology assistance, and paramedicine. A corresponding review question was established to guide this inquiry: What are the specific learning needs of students of pediatric nursing, midwifery, physiotherapy, occupational therapy, radiology assistance, and paramedicine?

### Stage 2: Identification of Relevant Studies

This review is being conducted in 3 sequential steps:

- Preliminary search: an initial search was performed in the PubMed database to inform the final search strategy.
- Comprehensive search: a thorough search was conducted across multiple databases to ensure extensive coverage of pertinent literature. The search was conducted in PubMed, Scopus, CINAHL Plus with Full Text, APA PsycInfo and APA PsycArticles, Academic Search Ultimate, Open Dissertations, Web of Science Core Collection, ProQuest Dissertations & Theses, Emcare, and Embase databases. The search has not been further limited by time or availability.
- Reference screening: the reference lists of selected studies will be examined to identify any supplementary sources.

### Stage 3: Study Selection Process

To ensure the selection of appropriate keywords, specific inclusion and exclusion criteria have been established (Textbox 1).

**Inclusion and exclusion criteria.**

**Inclusion criteria**

- University students enrolled in pediatric nursing, midwifery, physiotherapy, occupational therapy, and radiology assistance programs
- Individuals belonging to Generation Z (ie, born between 1995 and 2010)
- Focus on learning preferences and needs
- Papers in English
- All publication years
- All study designs

**Exclusion criteria**

- University students enrolled in medicine, general nursing, pharmacy, dentistry, or other non–health care programs
- High school students
- Individuals from Generation X, Generation Y, or Generation Alpha

These criteria are designed to ensure the review accurately reflects the learning preferences and needs of Gen Z university students within the specified health care disciplines. Exclusion of medicine and general nursing was based on the substantial existing body of literature addressing educational needs in these disciplines, in contrast to limited evidence from other health professions, such as midwifery, physiotherapy, occupational therapy, radiology assistance, paramedicine, and pediatric nursing. Therefore, this review intentionally focused on these specific disciplines to explore potential gaps in the literature. The generational timeframe was supported by the rationale to identify studies exploring an existing population in academia, hence the upper limit of 2010 was deemed sufficient. The selected keywords and searches in all databases are displayed in Tables 1-9 and Textbox 2.

**Table 1:** Keywords from the search in the PubMed database.

Set	Keywords
1	“Radiologists”[Mesh] OR “Nurses”[Mesh] OR “Nurses, Pediatric”[Mesh] OR “Paramedics”[Mesh] OR “Emergency Medical Technicians”[Mesh] OR “Midwifery”[Mesh] OR “Physical Therapists”[Mesh] OR “Allied Health Occupations”[Mesh] OR “Health Personnel”[Mesh] OR “Nurses, Neonatal”[Mesh] OR “Obstetric Nursing”[Mesh] OR paramedic*[Title/Abstract] OR “obstetric nurse*”[Title/Abstract] OR “neonatal nurse*”[Title/Abstract] OR “maternity nurse*”[Title/Abstract] OR “labour and delivery nurse*”[Title/Abstract] OR “labor and delivery nurse*”[Title/Abstract] OR “physical therapist*”[Title/Abstract] OR “radiology assistant*”[Title/Abstract] OR “radiology physician assistant*”[Title/Abstract] OR “emergency ambulance worker*”[Title/Abstract] OR “emergency medical service*”[Title/Abstract] OR radiologist*[Title/Abstract] OR ambulance*[Title/Abstract] OR “emergency medical technician*”[Title/Abstract] OR “pediatric nurs*”[Title/Abstract] OR “paediatric nurs*”[Title/Abstract] OR “children nurs*”[Title/Abstract] OR physiotherap*[Title/Abstract] OR midwife*[Title/Abstract] OR midwive*[Title/Abstract] OR “health professional*”[Title/Abstract] OR “allied health professional*”[Title/Abstract] OR “healthcare professional*”[Title/Abstract] OR “health personnel*”[Title/Abstract] OR “healthcare personnel*”[Title/Abstract] OR nurse?[Title/Abstract] OR nursing[Title/Abstract]
2	“Students”[Mesh] OR student*[Title/Abstract] OR learner? [Title/Abstract]
3	“Generation z”[Text Word] OR “gen z”[Text Word] OR igen[Text Word] OR “post-millennial*”[Text Word] OR “post millennial*”[Text Word] OR “postmillennial*”[Text Word]
4	“Education”[Mesh] OR “Learning”[Mesh] OR “Teaching”[Mesh] OR education*[Title/Abstract] OR educate[Title/Abstract] OR learn*[Title/Abstract] OR teaching[Title/Abstract] OR training[Title/Abstract] OR tutoring[Title/Abstract] OR instruction*[Title/Abstract] OR competen*[Title/Abstract] OR preference*[Title/Abstract] OR need [Title/Abstract] OR needs [Title/Abstract] OR ““values of life“ [Title/Abstract] OR “soft skill*“ [Title/Abstract] OR ““transversal skill”* [Title/Abstract]
5	#1 AND #2 AND #3 AND #4

**Table 2:** Keywords from SocINDEX with full text (EBSCOhost).

Set	Keywords
1	DE “NURSES” [Descriptor] OR DE “NURSING” [Descriptor] OR DE “MIDWIFERY” [Descriptor] OR DE “ALLIED health personnel” [Descriptor] OR paramedic* [Title/Abstract] OR obstetric nurse*”[Title/Abstract] OR “neonatal nurse*”[Title/Abstract] OR “maternity nurse*”[Title/Abstract] OR “labour and delivery nurse*”[Title/Abstract] OR “labor and delivery nurse*”[Title/Abstract] OR “physical therapist*”[Title/Abstract] OR “radiology assistant*”[Title/Abstract] OR “radiology physician assistant*”[Title/Abstract] OR “emergency ambulance worker*”[Title/Abstract] OR “emergency medical service*” [Title/Abstract] OR “ambulance officer*” [Title/Abstract] OR “emergency medical technician*” [Title/Abstract] OR “pediatric nurs*” [Title/Abstract] OR “children nurs*” [Title/Abstract] OR physiotherap* [Title/Abstract] OR midwife* [Title/Abstract] OR midwive* [Title/Abstract] OR “health professional*” [Title/Abstract] OR “healthcare professional*” [Title/Abstract] OR “health personnel*” [Title/Abstract] OR “allied health professional*”[Title/Abstract] OR “healthcare personnel*” [Title/Abstract] OR radiologist* [Title/Abstract] OR nurse [Title/Abstract] OR nurses [Title/Abstract] OR nursing [Title/Abstract]
2	DE “STUDENTS” [Descriptor] OR student* [Title/Abstract] OR learner? [Title/Abstract]
3	DE “GENERATION Z” [Descriptor] OR “generation z” [Text Word] OR “gen z” [Text Word] OR igen [Text Word] OR “post-millennial*” [Text Word] OR “post millennial*” [Text Word] OR “postmillennial*” [Text Word]
4	DE “TEACHING” [Descriptor] OR DE “LEARNING” [Descriptor] OR DE “EDUCATION” [Descriptor] OR education* [Title/Abstract] OR educate [Title/Abstract] OR learning [Title/Abstract] OR teaching [Title/Abstract] OR training [Title/Abstract] OR tutoring [Title/Abstract] OR instruction* [Title/Abstract] OR competen* [Title/Abstract] OR preference* [Title/Abstract] OR need [Title/Abstract] OR needs [Title/Abstract] OR ““values of life“ [Title/Abstract] OR ““soft skill*” [Title/Abstract] OR ““transversal skill*“ [Title/Abstract]
5	#1 AND #2 AND #3 AND #4

**Table 3:** Keywords from the APA PsycInfo and APA PsycArticles (EBSCOhost) databases.

Set	Keywords
1	DE “NURSES” [Descriptor] OR DE “NURSING” [Descriptor] OR DE “MIDWIFERY” [Descriptor] OR DE “ALLIED health personnel” [Descriptor] OR DE “Radiologists” [Descriptor] OR DE “Physical Therapy” [Descriptor] OR DE “Paramedics” [Descriptor] OR “paramedic*” [Title/Abstract] OR “obstetric nurse*”[Title/Abstract] OR “neonatal nurse*”[Title/Abstract] OR “maternity nurse*”[Title/Abstract] OR “labour and delivery nurse*”[Title/Abstract] OR “labor and delivery nurse*”[Title/Abstract] OR “physical therapist*”[Title/Abstract] OR “radiology assistant*”[Title/Abstract] OR “radiology physician assistant*”[Title/Abstract] OR “emergency ambulance worker*”[Title/Abstract] OR “emergency medical service*” [Title/Abstract] OR ambulance* [Title/Abstract] OR “emergency medical technician*” [Title/Abstract] OR “pediatric nurs*” [Title/Abstract] OR “children nurs*” [Title/Abstract] OR physiotherap* [Title/Abstract] OR midwife* [Title/Abstract] OR midwive* [Title/Abstract] OR “health professional*” [Title/Abstract] OR “healthcare professional*” [Title/Abstract] OR “health personnel*” [Title/Abstract] OR “healthcare personnel*” [Title/Abstract] OR “allied health professional*”[Title/Abstract] OR radiologist* [Title/Abstract] OR nurse [Title/Abstract] OR nurses [Title/Abstract] OR nursing [Title/Abstract]
2	DE “STUDENTS” [Descriptor] OR student* [Title/Abstract] OR learner? [Title/Abstract]
3	“generation z” [Text Word] OR “gen z” [Text Word] OR igen [Text Word] OR “post-millennial*” [Text Word] OR “post millennial*” [Text Word] OR “postmillennial*” [Text Word]
4	DE “Education” [Descriptor] OR DE “Learning” [Descriptor] OR DE “Teaching” [Descriptor] OR DE “Tutoring” [Descriptor] OR education* [Title/Abstract] OR educate [Title/Abstract] OR learning [Title/Abstract] OR teaching [Title/Abstract] OR training [Title/Abstract] OR tutoring [Title/Abstract] OR instruction* [Title/Abstract] OR competen* [Title/Abstract] OR preference* [Title/Abstract] OR need [Title/Abstract] OR needs [Title/Abstract] OR ““values of life” [Title/Abstract] OR ““soft skill*“ [Title/Abstract] OR “ “transversal skill*” [Title/Abstract]
5	#1 AND #2 AND #3 AND #4

**Table 4:** Keywords from the Academic Search Ultimate (EBSCOhost) database.

Set	Keywords
1	DE “PHYSICAL therapy” [Descriptor] OR DE “RADIOLOGISTS” [Descriptor] OR DE “NURSES” [Descriptor] OR DE “NURSING” [Descriptor] OR DE “MIDWIVES” [Descriptor] OR DE “PEDIATRIC nurses” [Descriptor] OR DE “ALLIED health personnel” [Descriptor] OR DE “EMERGENCY medical personnel” [Descriptor] OR DE “EMERGENCY medical technicians” [Descriptor] OR DE “NEONATAL nursing” [Descriptor] OR DE “MATERNITY nursing” [Descriptor] OR paramedic* [Title/Abstract] OR “emergency medical service*” [Title/Abstract] OR “ambulance officer”* [Title/Abstract] OR “emergency medical technician*” [Title/Abstract] OR “pediatric nurs*” [Title/Abstract] OR “paediatric nurs*” [Title/Abstract] OR “obstetric nurse*”[Title/Abstract] OR “neonatal nurse*”[Title/Abstract] OR “maternity nurse*”[Title/Abstract] OR “labour and delivery nurse*”[Title/Abstract] OR “labor and delivery nurse*”[Title/Abstract] OR “physical therapist*”[Title/Abstract] OR “radiology assistant*”[Title/Abstract] OR “radiology physician assistant*”[Title/Abstract] OR “emergency ambulance worker*”[Title/Abstract] “children nurs*” [Title/Abstract] OR physiotherap* [Title/Abstract] OR midwife* [Title/Abstract] OR midwive* [Title/Abstract] OR “health professional*” [Title/Abstract] OR “healthcare professional*” [Title/Abstract] OR “allied health professional*”[Title/Abstract] OR “health personnel*” [Title/Abstract] OR “healthcare personnel*” [Title/Abstract] OR radiologist* [Title/Abstract] OR nurse [Title/Abstract] OR nurses [Title/Abstract] OR nursing [Title/Abstract]
2	DE “STUDENTS” [Descriptor] OR student* [Title/Abstract] OR learner? [Title/Abstract]
3	DE “GENERATION Z” [Descriptor] OR “generation z” [Text Word] OR “gen z” [Text Word] OR igen [Text Word] OR “post-millennial*” [Text Word] OR “post millennial*” [Text Word] OR “postmillennial*”) [Text Word]
4	DE “TEACHING” [Descriptor] OR DE “LEARNING” [Descriptor] OR DE “TUTORS & tutoring” [Descriptor] OR DE “EDUCATION” [Descriptor] OR education* [Title/Abstract] OR educate [Title/Abstract] OR learn* [Title/Abstract] OR teaching [Title/Abstract] OR training [Title/Abstract] OR tutoring [Title/Abstract] OR instruction* [Title/Abstract] OR competen* [Title/Abstract] OR preference* [Title/Abstract] OR need [Title/Abstract] OR needs [Title/Abstract] OR ““values of life” [Title/Abstract] OR ““soft skill*” [Title/Abstract] OR ““transversal skill*” [Title/Abstract]
5	#1 AND #2 AND #3 AND #4

**Table 5:** Keywords from the OpenDissertations (EBSCOhost) database.

Set	Keywords
1	Paramedic* [Title/Abstract] OR “obstetric nurse*”[Title/Abstract] OR “neonatal nurse*”[Title/Abstract] OR “maternity nurse*”[Title/Abstract] OR “labour and delivery nurse*”[Title/Abstract] OR “labor and delivery nurse*”[Title/Abstract] OR “physical therapist*”[Title/Abstract] OR “radiology assistant*”[Title/Abstract] OR “radiology physician assistant*”[Title/Abstract] OR “emergency ambulance worker*”[Title/Abstract] OR “emergency medical service*” [Title/Abstract] OR ambulance* [Title/Abstract] OR “emergency medical technician*” [Title/Abstract] OR “pediatric nurs*” [Title/Abstract] OR “paediatric nurse*” [Title/Abstract] OR “children nurs*” [Title/Abstract] OR physiotherap* [Title/Abstract] OR midwife* [Title/Abstract] OR midwive* [Title/Abstract] OR “health professional*” [Title/Abstract] OR “healthcare professional*” [Title/Abstract] OR “health personnel*” [Title/Abstract] OR “healthcare personnel*” [Title/Abstract] OR “allied health professional*”[Title/Abstract] OR radiologist* [Title/Abstract] OR nurse [Title/Abstract] OR nurses [Title/Abstract] OR nursing [Title/Abstract]
2	student* [Title/Abstract] OR learner? [Title/Abstract]
3	“generation z” [Text Word] OR “gen z” [Text Word] OR igen [Text Word] OR “post-millennial*” [Text Word] OR “post millennial*” [Text Word] OR “postmillennial*” [Text Word]
4	education* [Title/Abstract] OR educate [Title/Abstract] OR learning [Title/Abstract] OR teaching [Title/Abstract] OR training [Title/Abstract] OR tutoring [Title/Abstract] OR instruction* [Title/Abstract] OR competen* [Title/Abstract] OR preference* [Title/Abstract] OR need [Title/Abstract] OR needs [Title/Abstract] OR “values of life” [Title/Abstract] OR “soft skill*” [Title/Abstract] OR “transversal skill*” [Title/Abstract]
5	#1 AND #2 AND #3 AND #4

**Table 6:** Keywords from the Web of Science Core Collection (Clarivate Analytics) database.

Set	Keywords
1	paramedic* [Title/Abstract] OR “obstetric nurse*”[Title/Abstract] OR “neonatal nurse*”[Title/Abstract] OR “maternity nurse*”[Title/Abstract] OR “labour and delivery nurse*”[Title/Abstract] OR “labor and delivery nurse*”[Title/Abstract] OR “physical therapist*”[Title/Abstract] OR “radiology assistant*”[Title/Abstract] OR “radiology physician assistant*”[Title/Abstract] OR “emergency ambulance worker*”[Title/Abstract] OR “emergency medical service*” [Title/Abstract] OR ambulance* [Title/Abstract] OR “emergency medical technician*” [Title/Abstract] OR “pediatric nurs*” [Title/Abstract] OR “paediatric nurse*” [Title/Abstract] OR “children nurs*” [Title/Abstract] OR physiotherap* [Title/Abstract] OR midwife* [Title/Abstract] OR midwive* [Title/Abstract] OR “health professional*” [Title/Abstract] OR “healthcare professional*” [Title/Abstract] OR “health personnel*” [Title/Abstract] OR “healthcare personnel*” [Title/Abstract] OR “allied health professional*”[Title/Abstract] OR radiologist* [Title/Abstract] OR nurse [Title/Abstract] OR nurses [Title/Abstract] OR nursing [Title/Abstract]
2	student* [Title/Abstract] OR learner? [Title/Abstract]
3	“generation z” [All fields] OR “gen z” [All fields] OR igen [All fields] OR “post-millennial*” [All fields] OR “post millennial*” [All fields] OR “postmillennial*” [All fields]
4	education* [Title/Abstract] OR educate [Title/Abstract] OR learning [Title/Abstract] OR teaching [Title/Abstract] OR training [Title/Abstract] OR tutoring [Title/Abstract] OR instruction* [Title/Abstract] OR competen* [Title/Abstract] OR preference* [Title/Abstract] OR need [Title/Abstract] OR needs [Title/Abstract] OR values of life [Title/Abstract] OR soft skill* [Title/Abstract] OR transversal skill* [Title/Abstract]
5	#1 AND #2 AND #3 AND #4

**Table 7:** Keywords from ProQuest Dissertations & Theses (Clarivate Analytics).

Set	Keywords
1	Paramedic* [Title/Abstract] OR “obstetric nurse*”[Title/Abstract] OR “neonatal nurse*”[Title/Abstract] OR “maternity nurse*”[Title/Abstract] OR “labour and delivery nurse*”[Title/Abstract] OR “labor and delivery nurse*”[Title/Abstract] OR “physical therapist*”[Title/Abstract] OR “radiology assistant*”[Title/Abstract] OR “radiology physician assistant*”[Title/Abstract] OR “emergency ambulance worker*”[Title/Abstract] OR “emergency medical service*” [Title/Abstract] OR ambulance* [Title/Abstract] OR “emergency medical technician*” [Title/Abstract] OR “pediatric nurs*” [Title/Abstract] OR “paediatric nurs*” [Title/Abstract] OR “children nurs*” [Title/Abstract] OR physiotherap* [Title/Abstract] OR midwife* [Title/Abstract] OR midwive* [Title/Abstract] OR “health professional*” [Title/Abstract] OR “healthcare professional*” [Title/Abstract] OR “health personnel*” [Title/Abstract] OR “healthcare personnel*” [Title/Abstract] OR “allied health professional*”[Title/Abstract] OR radiologist* [Title/Abstract] OR nurse [Title/Abstract] OR nurses [Title/Abstract] OR nursing [Title/Abstract]
2	student* [Title/Abstract] OR learner?
3	“generation z” [Title/Abstract] OR “gen z” [Title/Abstract] OR igen [Title/Abstract] OR “post-millennial*” [Title/Abstract] OR “post millennial*” [Title/Abstract] OR “postmillennial*” [Title/Abstract]
4	education* [Title/Abstract] OR educate [Title/Abstract] OR learning [Title/Abstract] OR teaching [Title/Abstract] OR training [Titte/Abstract] OR tutoring [Title/Abstract] OR instruction* [Title/Abstract] OR competen* [Title/Abstract] OR preference* [Title/Abstract] OR need [Title/Abstract] OR needs [Title/Abstract] OR “values of life” [Title/Abstract] OR “soft skill*” [Title/Abstract] OR “transversal skill*” [Title/Abstract]
5	#1 AND #2 AND #3 AND #4

**Table 8:** Keywords from the Scopus (Elsevier) database.

Set	Keywords
1	paramedic* [Title/Abstract] OR “obstetric nurse*”[Title/Abstract] OR “neonatal nurse*”[Title/Abstract] OR “maternity nurse*”[Title/Abstract] OR “labour and delivery nurse*”[Title/Abstract] OR “labor and delivery nurse*”[Title/Abstract] OR “physical therapist*”[Title/Abstract] OR “radiology assistant*”[Title/Abstract] OR “radiology physician assistant*”[Title/Abstract] OR “emergency ambulance worker*”[Title/Abstract] OR “emergency medical service*” [Title/Abstract] OR ambulance* [Title/Abstract] OR “emergency medical technician*” [Title/Abstract] OR “pediatric nurs*” [Title/Abstract] OR “paediatric nurs*” [Title/Abstract] OR “children nurs*” [Title/Abstract] OR physiotherap* [Title/Abstract] OR midwife* [Title/Abstract] OR midwive* [Title/Abstract] OR “health professional*” [Title/Abstract] OR “healthcare professional*” [Title/Abstract] OR “health personnel*” [Title/Abstract] OR “healthcare personnel*” [Title/Abstract] OR “allied health professional*”[Title/Abstract] OR radiologist* [Title/Abstract] OR nurse [Title/Abstract] OR nurses [Title/Abstract] OR nursing [Title/Abstract]
2	student* [Title/Abstract] OR learner?
3	“generation z” [All fields] OR “gen z” [All fields] OR igen [All fields] OR “post-millennial*” [All fields] OR “post millennial*” [All fields] OR “postmillennial*” [All fields]
4	education* [Title/Abstract] OR educate [Title/Abstract] OR learning [Title/Abstract] OR teaching [Title/Abstract] OR training [Titte/Abstract] OR tutoring [Title/Abstract] OR instruction* [Title/Abstract] OR competen* [Title/Abstract] OR preference* [Title/Abstract] OR need [Title/Abstract] OR needs [Title/Abstract] OR “values of life“ [Title/Abstract] OR “soft skill*” [Title/Abstract] OR “transversal skill*” [Title/Abstract]
5	#1 AND #2 AND #3 AND #4

**Table 9:** Keywords from the Embase and Emcare databases.

Set	Keywords
1	radiologist/
2	nurse/
3	pediatric nurse/
4	paramedical personnel/
5	rescue personnel/
6	midwife/
7	physiotherapist/
8	health care personnel/
9	neonatal nurse/
10	obstetrical nursing/
11	(paramedic OR “obstetric nurse*” OR “neonatal nurse*” OR “maternity nurse*” OR “labor and delivery nurse*” OR “physical therapist*” OR “radiology assistant*” OR “radiology physician assistant*” OR “emergency ambulance worker*” OR “emergency medical service*” OR radiologist* OR ambulance* OR “emergency medical technician*” OR “pediatric nurs*” OR “children nurs*” OR physiotherap* OR midwife* OR midwive* OR “health professional*” OR “allied health professional*” OR “healthcare professional*OR health personnel*” OR “healthcare personnel*” OR nurse*).ti,ab.
12	1 OR 2 OR 3 OR 4 OR 5 OR 6 OR 7 OR 8 OR 9 OR 10 OR 11
13	student/
14	(student* OR learner?).ti,ab.
15	13 OR 14
16	(“Generation z” OR “gen z” OR igen OR “post-millennial*” OR “post millennial*” OR “postmillennial*”).mp. [mp=title, abstract, heading word, drug trade name, original title, device manufacturer, drug manufacturer, device trade name, keyword heading word, floating subheading word, candidate term word]
17	education/
18	learning/
19	teaching/
20	(education* OR educate or learn* OR teaching or training or tutoring or instruction* OR competen* OR preference* OR need* OR “values of life” OR “soft skill*” OR “transversal skill*”).ti,ab.
21	17 OR 18 OR 19 OR 20
22	12 AND 15 AND 16 AND 21

**Keywords from Google Scholar (Google).**

(health care student OR midwifery student OR radiologist student OR physiotherapist OR pediatric nurse student OR nurse student OR nursing student OR paramedic student) AND (generation Z OR gen Z OR igen OR postmillennial) AND (education OR educate OR teaching OR learning OR tutoring OR preference OR need OR instruction)

The screening process was conducted by a broad team of researchers to ensure diverse clinical and academic backgrounds and experience. The steps in study selection involved:

- Initial screening: review of titles and abstracts to identify relevant studies
- Full-text assessment: evaluation of full texts of selected studies for eligibility
- References: screening of reference lists of included papers to identify additional pertinent studies

**Figure 1:**
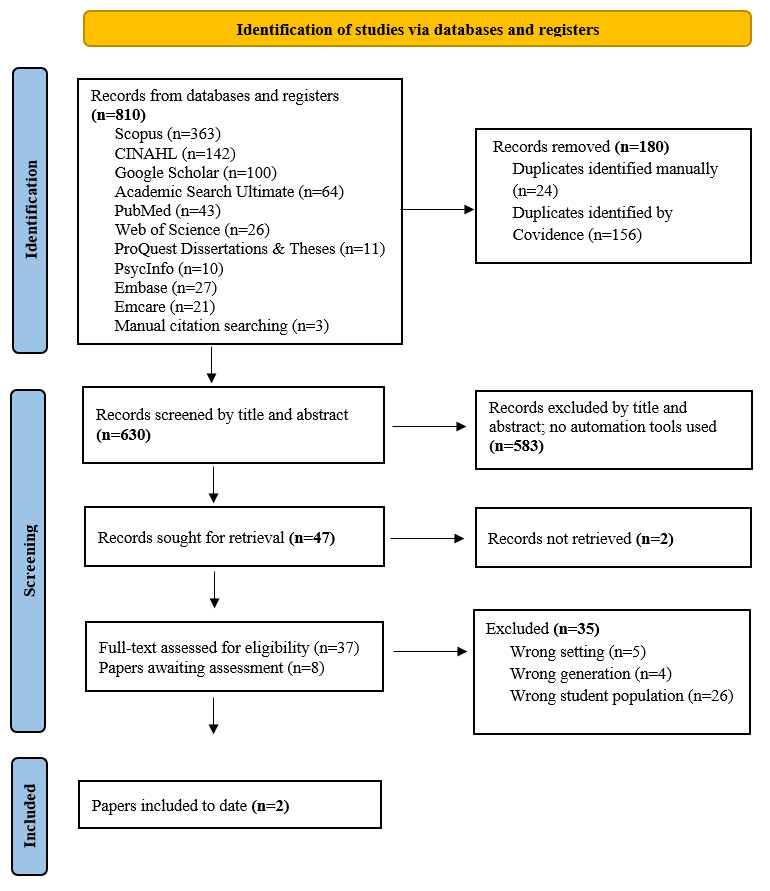
PRISMA (Preferred Reporting Items for Systematic Reviews and Meta-Analyses) flow diagram of study selection to date.

This process adheres to the Joanna Briggs Institute (JBI) guidelines. Covidence software (Veritas Health Innovation) has been used for study screening and selection. The entire search procedure has been documented using a PRISMA (Preferred Reporting Items for Systematic Reviews and Meta-Analyses) flow diagram (Figure 1). Any disagreements or ambiguities were resolved through discussions among the research team, supported by 4 senior researchers. Titles and abstracts were screened in Covidence independently by 2 teams of 2 researchers (IV, EP, PK, and LV), holding regular meetings with a fifth researcher (DJ) overseeing screening for consistency. Full-text screening was conducted independently by 2 reviewers (LV and DJ) with a third researcher resolving conflicts (ORA), and the expert team is available for any further discussion if needed. In cases where studies included mixed age groups or multiple disciplines, and/or it was not possible to extract data specifically for Gen Z students or the selected study programs, such studies were excluded from the review.

### Stage 4: Data Extraction

Relevant data will be extracted from full-text papers by a research team of doctoral students with the supervision of 4 senior researchers. Two reviewers (LV and ORA) will independently pilot and then perform the data extraction using a form designed in Microsoft Excel. The data extraction will follow the JBI guidelines for scoping reviews. Critical appraisal will not be conducted, as the aim of this scoping review is to map the available evidence rather than assess the methodological quality of individual studies, in line with JBI and PRISMA-ScR guidance.

Both quantitative and qualitative data will be analyzed to identify themes and patterns relevant to the learning needs of Gen Z students in pediatric nursing, midwifery, physiotherapy, occupational therapy, radiology assistance, and paramedicine. Data extraction will include study characteristics (author, year, country, and study objective), methodology, description of the target population (sample size and age), and key findings regarding learning preferences and characteristics. Specific items will also encompass learning modalities, technology use, and assessment preferences. Both qualitative and quantitative findings will be synthesized narratively, integrating themes and patterns across studies to provide a comprehensive overview of Gen Z students’ learning characteristics in pediatric nursing, midwifery, and allied health professions. Data extraction will ensure consistency and transparency by adhering to predefined categories while also allowing the identification of unanticipated issues.

The outcomes of data extraction will be presented in tabular as well as narrative form. The age range identified during full-text screening will be recorded in the data extraction sheet.

### Stage 5: Collating, Synthesizing, and Reporting Results

The analysis process for this scoping review will involve systematic methods to extract, chart, and summarize data from included studies.

The synthesis will aim to integrate data from multiple studies into a cohesive narrative. Findings will be mapped against key domains of learning needs, such as learning styles, technological preferences, support mechanisms, and barriers to engagement in classrooms. Results will be categorized based on overarching themes (eg, personalized learning, social connection, and other relevant domains).

## Results

The initial search was conducted on December 18, 2024, across the Scopus, CINAHL, Google Scholar, PubMed, Web of Science, PsycInfo, ProQuest Dissertations & Theses, and Academic Search Ultimate databases by a librarian at Palacký University Olomouc. The study was funded in December 2024. The combined search retrieved 759 records. After removing duplicates in Covidence and through manual checking, 590 (77.7%) unique records remained for title and abstract screening. Of these, 37 (6.3%) articles were selected for full-text review, resulting in the inclusion of 2 (5.4%) studies.

Given the limited number of eligible studies, the research team decided to expand the search strategy to include 2 additional databases. The OSF record was updated on June 2, 2025. An extended search in the Embase and Emcare databases was performed on July 28, 2025, by a King Edward Memorial Hospital librarian, yielding 51 records. Following duplicate removal (n=13, 25.5%), 38 (74.5%) unique records underwent title and abstract screening. At the time of reporting, the research team will review the full texts of 8 (21.1%) studies in December 2025, followed by screening the reference lists for additional eligible articles. Once agreement is reached, data extraction and synthesis are to follow. The results are expected to be published by the end of July 2026.

## Discussion

This scoping review is being conducted in accordance with the PRISMA-ScR guidelines and the JBI methodological framework to ensure a systematic, transparent, and replicable process aligned with international standards for evidence synthesis.

A comprehensive search strategy across 10 databases, including both peer-reviewed and gray literature, will allow for an extensive mapping of the existing evidence on the learning preferences of Gen Z students in selected health care disciplines. Screening the reference lists of included studies will further strengthen the coverage and help identify additional relevant sources.

A potential limitation of this review is its linguistic scope, restricted to English-language publications, which may result in the exclusion of studies published in other languages. Nevertheless, this decision balances feasibility with methodological consistency and ensures the accuracy of data interpretation.

Despite the search strategy, only a limited number of eligible studies were identified. This may reflect a genuine scarcity of research addressing Gen Z students of specific study programs. At the same time, it is possible that relevant studies were not retrieved due to variability in the use of generational labels. Many studies examine health care students’ learning experiences without explicitly identifying them as Gen Z, and age characteristics are often insufficiently reported. There is a possible limitation that relevant studies may not have been captured if generational identity was not specified in titles, abstracts, or keywords.

In addition to identifying and mapping current evidence, this review aims to provide a conceptual foundation for future empirical studies exploring how Gen Z health care students engage with technology, teamwork, and patient interaction during their education. The synthesis may highlight the need for innovative teaching strategies and institutional support systems that reflect this generation’s values and expectations. Ultimately, the findings are expected to inform curriculum development and research agendas focused on improving student engagement, retention, and readiness for clinical practice across health care disciplines.

## Data Availability

All data from this scoping review, including search strategies, screening decisions, and data extraction tables, will be made openly available via the Open Science Framework [34] upon publication of the final review.

## Abbreviations

Gen Z: Generation Z
JBI: Joanna Briggs Institute
OSF: Open Science Framework
PRISMA: Preferred Reporting Items for Systematic Reviews and Meta-Analyses
PRISMA-ScR: Preferred Reporting Items for Systematic Reviews and Meta-Analyses Extension for Scoping Reviews
